# Supplementary material for: Molecular and structural basis of the chromatin remodeling activity by Arabidopsis DDM1
Source: Nat Commun. 2024 Jul 11;15:5187. doi: 10.1038/s41467-024-49465-w (PMC11239853; doi:10.1038/s41467-024-49465-w)
Supplement: Supplementary file 1 — Supplementary Information [file 41467_2024_49465_MOESM1_ESM.pdf]

Supplementary Information for

Molecular and structural basis of the chromatin remodeling activity by *Arabidopsis* DDM1

Akihisa Osakabe[1,2#*], Yoshimasa Takizawa[3#], Naoki Horikoshi[3], Suguru Hatazawa[3], Lumi Negishi[3], Shoko Sato[3], Frédéric Berger[4], Tetsuji Kakutani[1*], Hitoshi Kurumizaka[1,3*]

[1]Department of Biological Sciences, Graduate School of Science, The University of Tokyo, Tokyo, Japan

[2]PRESTO, Japan Science and Technology Agency, Kawaguchi, Japan

[3]Laboratory of Chromatin Structure and Function, Institute for Quantitative Biosciences, The University of Tokyo, Tokyo, Japan

[4]Gregor Mendel Institute (GMI), Austrian Academy of Sciences, Vienna Biocenter (VBC), Vienna, Austria

[#]These two authors contributed equally to this work.

[*]Corresponding authors. E-mail: akihisa-osakabe@g.ecc.u-tokyo.ac.jp; tkak@bs.s.u-tokyo.ac.jp; kurumizaka@iqb.u-tokyo.ac.jp

Supplementary Table 1: Cryo-EM data collection, refinement, and validation statistics

Sample	DDM1-nucleosome complex (EMD-36083) (PDB: 8J90)		Nucleosome containing H2A.W (EMD-36085) (PDB: 8J92)	Nucleosome containing AtH2A (EMD-36084) (PDB: 8J91)
**Data collection**	Dataset 1	Dataset 2		
Elrctron microscope	Krios G4		Krios G4	Krios G4
Camera	K3		K3	K3
Pixel size (A/pix)	1.06		1.06	1.06
Decocus range (µm)	-1 to -2.5		-1 to -2.5	-1 to -2.5
Exposure time (second)	4.5		4.5	4.5
Total dose (e/A2)	63.5	60	59.1	59
Movie frames (no.)	40		40	40
Total micrographs (no.)	11,044	5,117	5,570	6,520
**Reconstruction**				
Software	Relion 4.0		Relion 4.0	Relion 4.0
Particles for 2D classification	7,004,935	2,125,419	4,254,793	3,651,924
Particles for 3D classification	895,881	898,969	2,579,646	2,141,126
Particles in the final map (no.)	34,559		196,430	411,432
Symmetry	C1		C1	C1
Final resolution (Å)	4.71		2.94	2.94
FSC threshold	0.143		0.143	0.143
Map sharpening B factor (Å2)	-160.4		-68.96	-95.9
**Model building**				
Software	Coot		Coot	Coot
**Refinement**				
Software	Phenix		Phenix	Phenix
**Model composition**				
Protein	1168		749	678
Nucleotide	222		298	226
**R.m.s deviations**				
Bond lengths (Å)	0.005		0.004	0.011
Bond angles (˚)	0.842		0.583	1.553
**Validation**				
MolProbity score	2.15		1.42	1.32
Clash score	22.93		7.73	5.81
**Ramachandran plot**				
Favored (%)	95.71		98.77	98.79
Allowed (%)	4.29		1.23	1.21
Outluers (%)	0		0	0

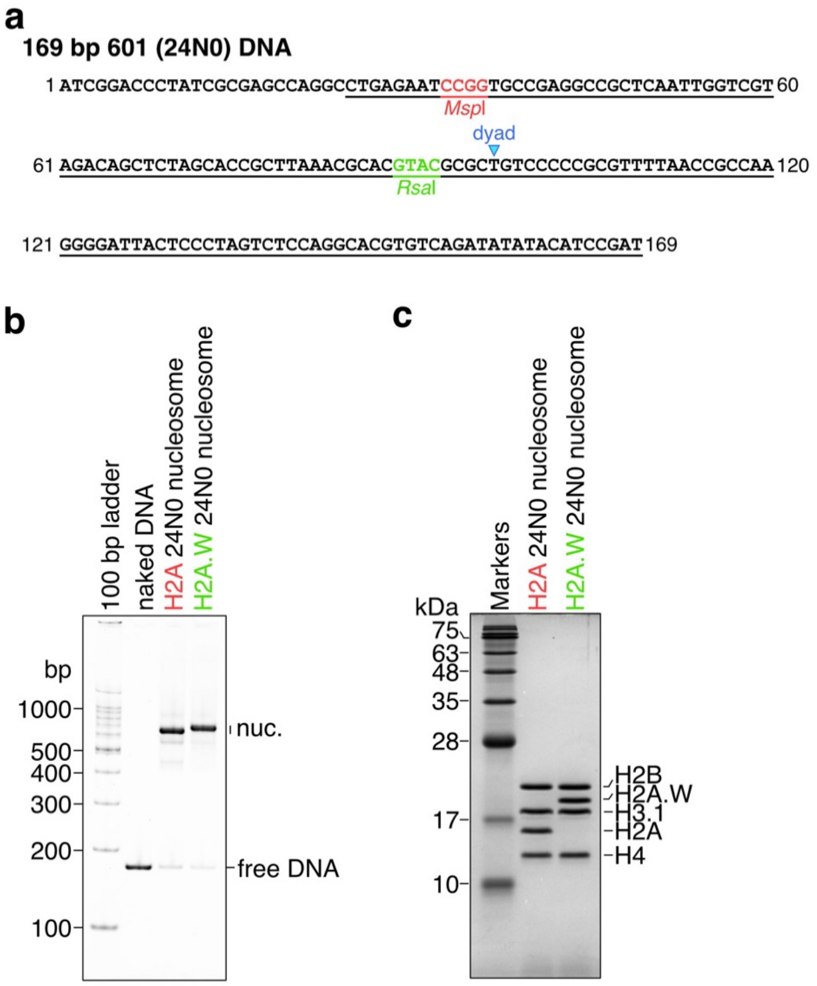

Supplementary Figure 1: Preparation of nucleosomes containing AtH2A and AtH2A.W.

a, Sequence of the DNA fragment used for nucleosome reconstitution. The Widom 601 positioning sequence is underlined. The blue arrowhead indicates the dyad axis. The *Msp*I and *Rsa*I recognition sites are colored red and green, respectively. **b and c,** Native-PAGE (**b**) and SDS-PAGE (**c**) analyses of purified nucleosomes containing AtH2A and AtH2A.W.

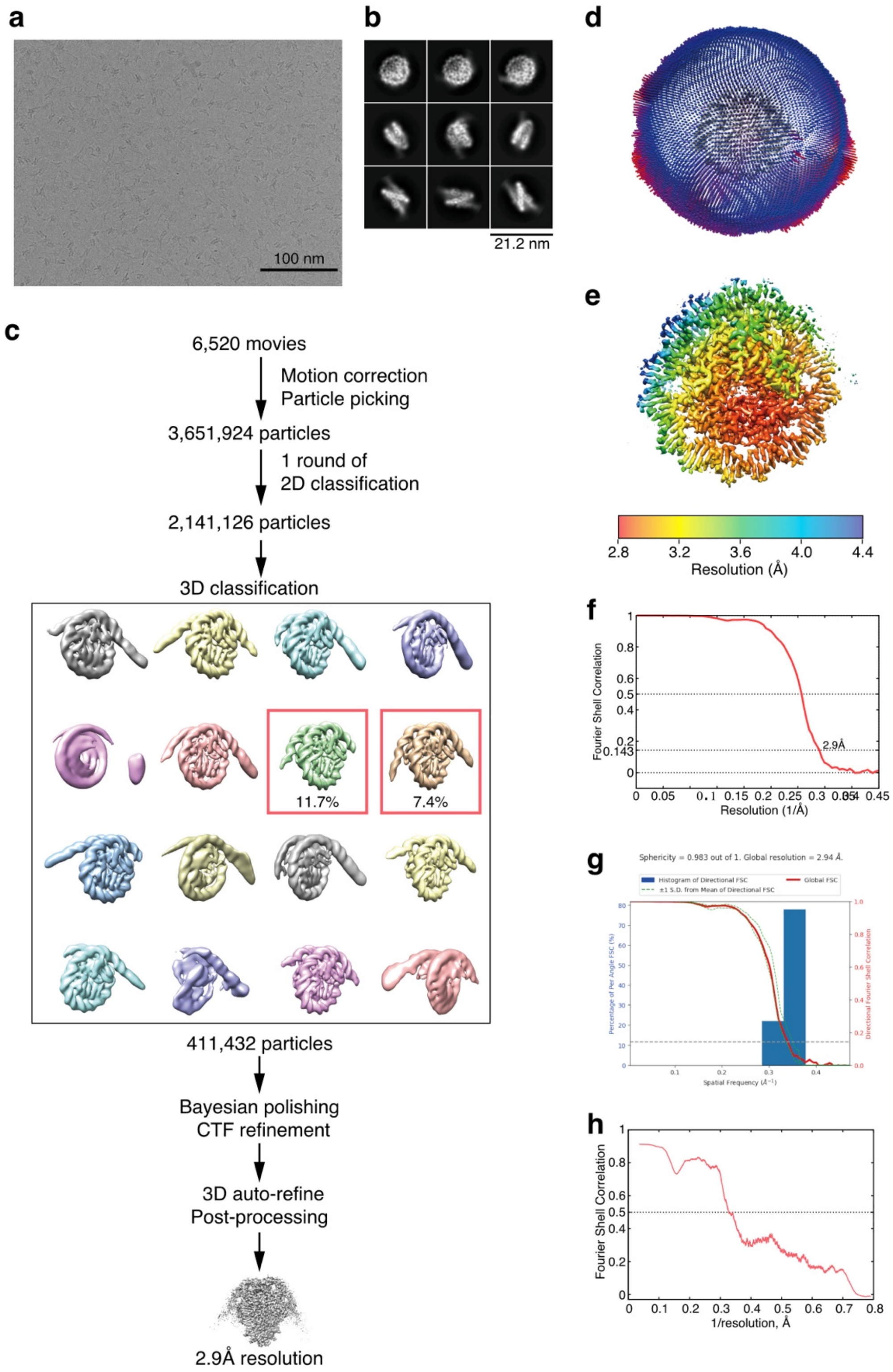

Supplementary Figure 2: Cryo-EM data collection and image processing of nucleosomes containing AtH2A.

a, Representative cryo-EM micrograph of the AtH2A nucleosome. Scale bar is 100 nm. **b,** Representative 2D class averages of AtH2A nucleosome. Box size is 21.2 nm. **c,** Flow

chart of the cryo-EM image processing of the AtH2A nucleosome. **d,** Euler angle distribution of the 3D reconstruction of the AtH2A nucleosome. **e,** Local resolution map of the AtH2A nucleosome. **f,** The "gold standard" Fourier Shell Correlation (FSC) curve of the AtH2A nucleosome, calculated between 3D reconstructions from two halves of data sets. **g,** 3D FSC curve for the reconstruction of the AtH2A nucleosome, calculated on the 3DFSC server[1] (https://3dfsc.salk.edu/upload/). **h,** Map-to-model FSC curve of AtH2A nucleosome calculated with Phenix.

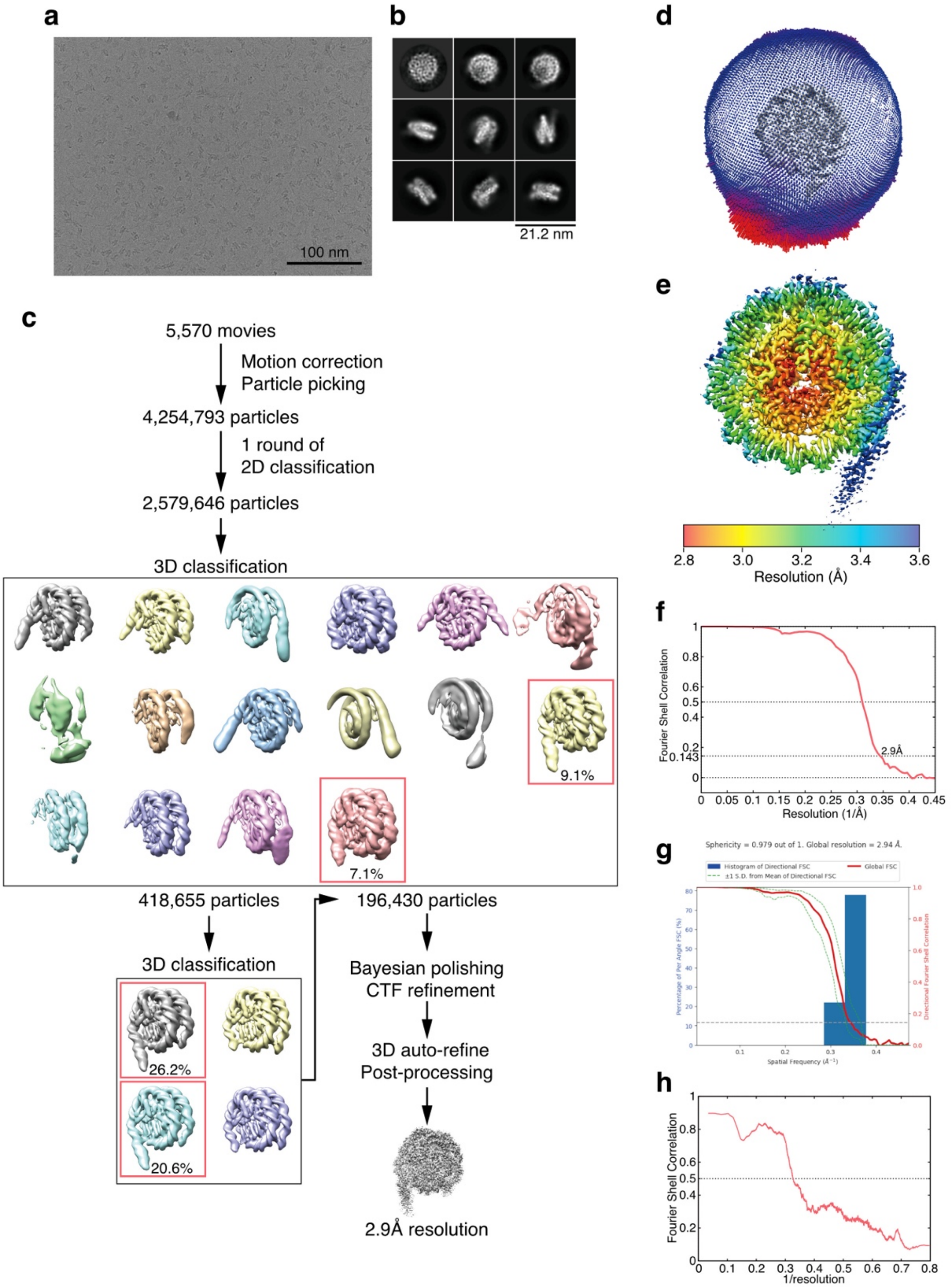

Supplementary Figure 3: Cryo-EM data collection and image processing of nucleosomes containing AtH2A.W.

a, Representative cryo-EM micrograph of the AtH2A.W nucleosome. Scale bar is 100 nm. **b,** Representative 2D class averages of the AtH2A.W nucleosome. Box size is 21.2 nm. **c,** Flow chart of the cryo-EM image processing of the AtH2A.W nucleosome. **d,** Euler angle distribution of the 3D reconstruction of the AtH2A.W nucleosome. **e,** Local

resolution map of AtH2A.W nucleosome. **f,** The "gold standard" Fourier Shell Correlation (FSC) curve of the AtH2A.W nucleosome, calculated between 3D reconstructions from two halves of data sets. **g,** 3D FSC curve for the reconstruction of the AtH2A.W nucleosome, calculated on the 3DFSC server[1] (https://3dfsc.salk.edu/upload/). **h,** Map-to-model FSC curve of AtH2A.W nucleosome calculated with Phenix.

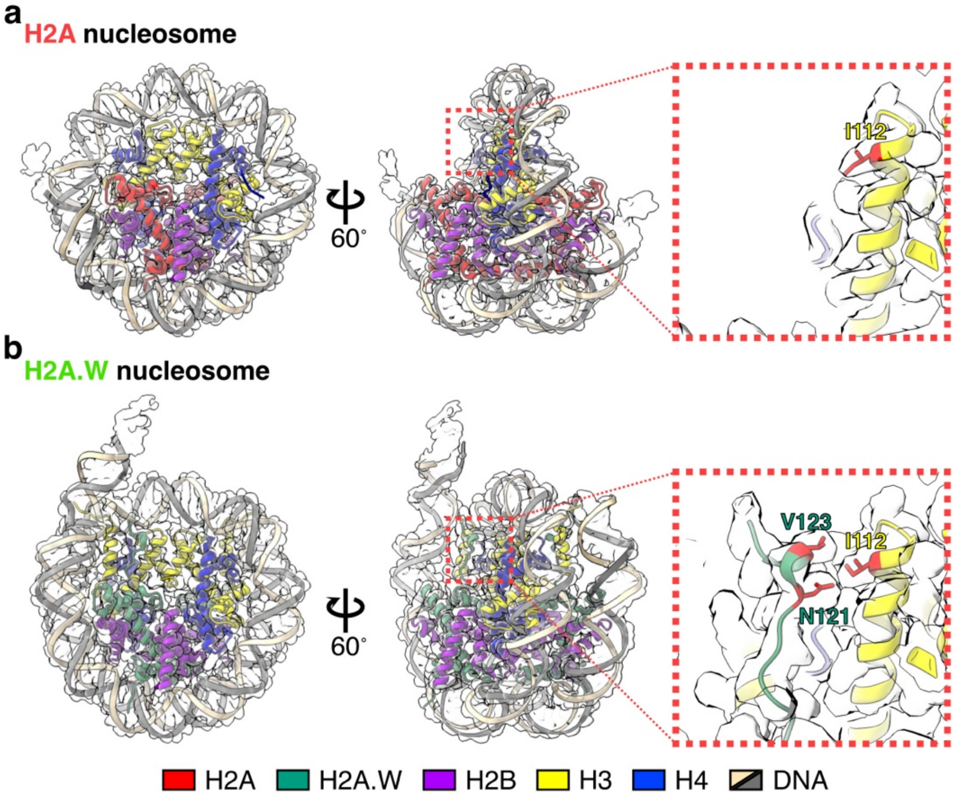

Supplementary Figure 4: Map to density figures of nucleosomes containing AtH2A and AtH2A.W.

The cryo-EM maps for the nucleosomes containing AtH2A (**a**) and AtH2A.W (**b**). Red dashed boxes indicate the interactions between the H2A.W docking domain and the H3 α2 helix.

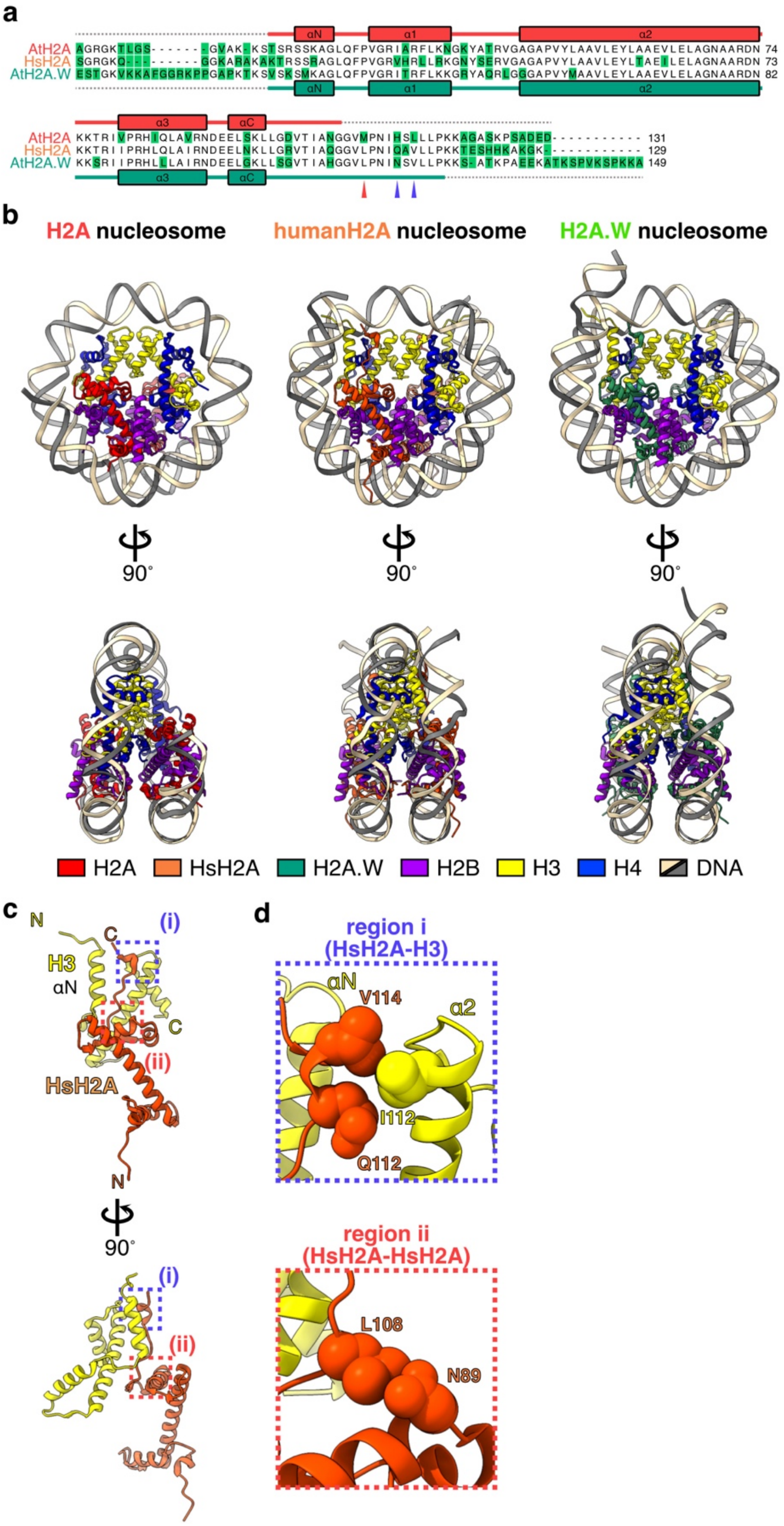

Supplementary Figure 5: Structural comparison of AtH2A, human H2A, and AtH2A.W nucleosomes.

a, Amino acid alignment of AtH2A (H2A.13: NP_188703.1), human H2A (H2AFA: NP_066390.1), and AtH2A.W (H2A.W.6: NP_200795.1). The secondary structures of AtH2A and AtH2A.W determined in this study are shown below and above the sequences, where the boxes and dashed lines indicate α helices and disordered regions, respectively. Red and blue arrowheads indicate the residues contacting the H3 α2 helix (Ile112) and the intra-protein interactions of H2A.W discussed in Figure 1e. **b,** Structures of the nucleosomes containing AtH2A (left), human H2A (middle) (PDB ID: 7VZ4 [https://www.rcsb.org/structure/7VZ4] (human nucleosome core particle)), and AtH2A.W (right). **c,** Structure of human H2A complexed with H3. The two contact sites discussed in panel (**d**) are enclosed in dashed boxes (i) and (ii). **d,** Close-up view of regions (i) and (ii) from panel (**c**), where human H2A and H3 are colored orange and yellow, respectively.

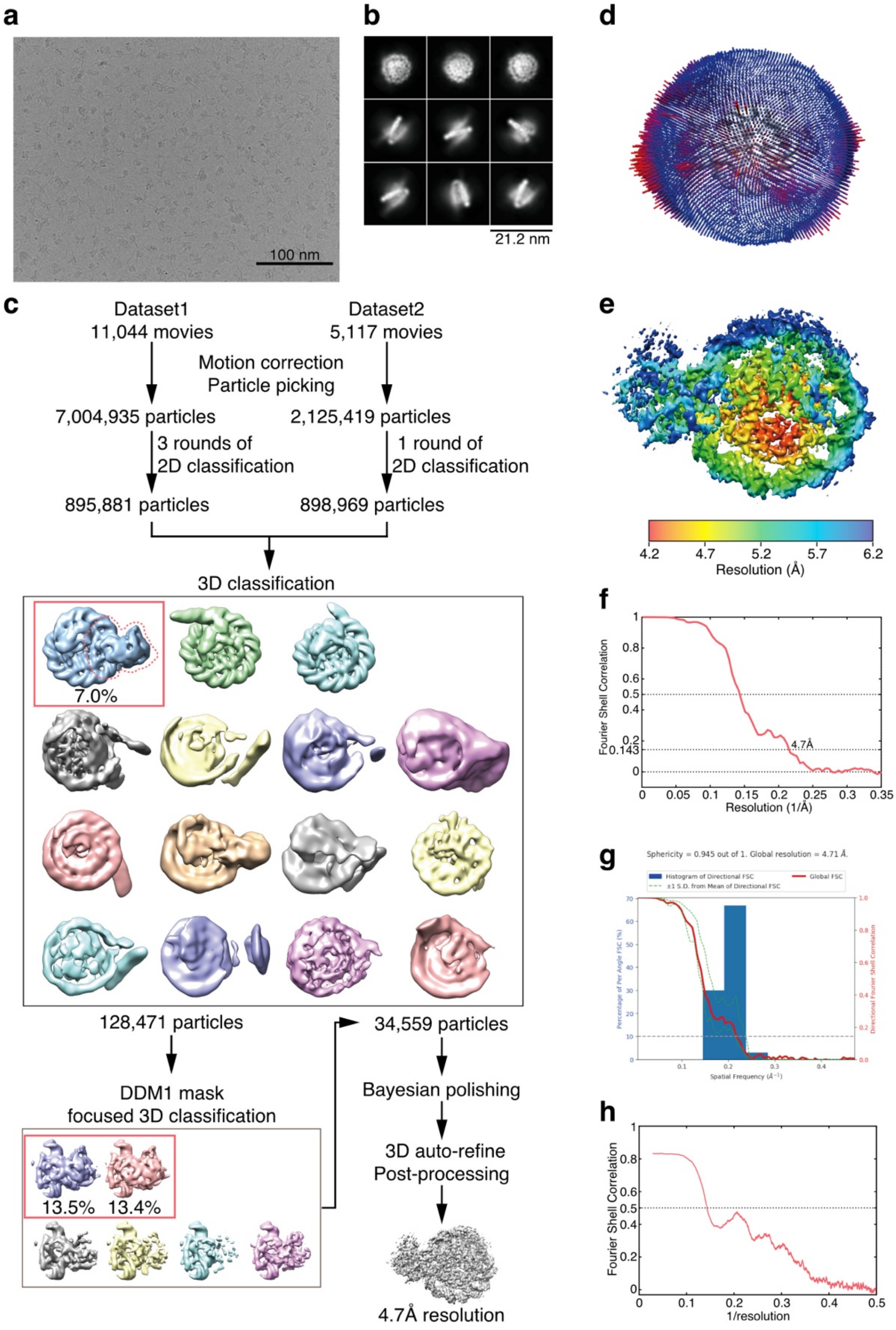

Supplementary Figure 6: Cryo-EM data collection and image processing of the AtDDM1-nucleosome complex.

a, Representative cryo-EM micrograph. Scale bar: 100 nm. **b,** Representative 2D class averages. Box size is 21.2 nm. **c,** Flow chart of the cryo-EM image processing of the AtDDM1-nucleosome complex. **d,** Euler angle distribution of the 3D reconstruction of the AtDDM1-nucleosome complex. **e,** Local resolution map of the AtDDM1-nucleosome complex. **f,** The "gold standard" Fourier Shell Correlation (FSC) curve of the AtDDM1-nucleosome complex, calculated between 3D reconstructions from two halves of data sets. **g,** 3D FSC curve for the reconstruction of the AtDDM1-nucleosome complex, calculated on the 3DFSC server[1] (https://3dfsc.salk.edu/upload/). **h,** Map-to-model FSC curve of the AtDDM1-nucleosome complex, calculated with Phenix.

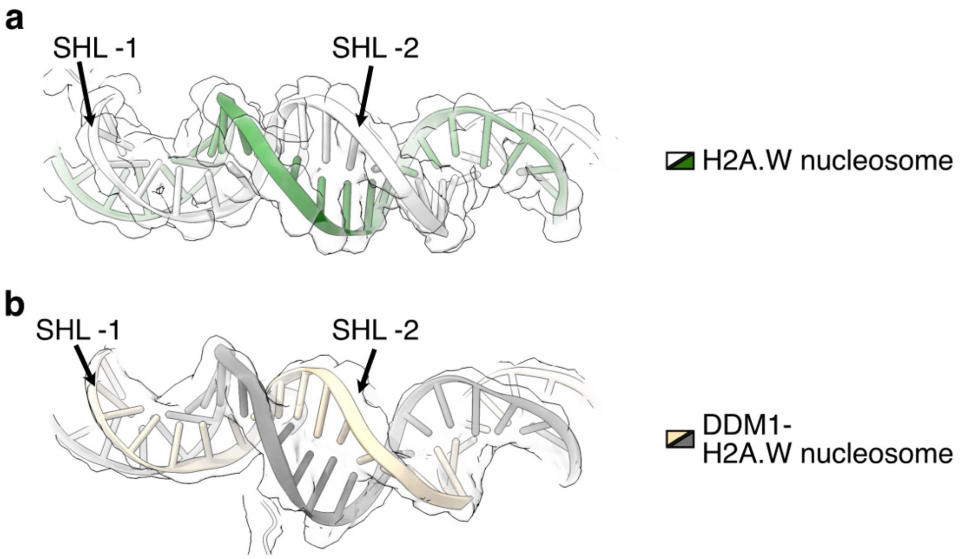

Supplementary Figure 7: Map to density figures of the AtH2A.W nucleosome and the AtDDM1-bound H2A.W nucleosome.

Cryo-EM maps for the nucleosomal DNA at SHL-2 of the AtH2A.W nucleosome (**a**) and the AtDDM1-AtH2A.W nucleosome complex (**b**).

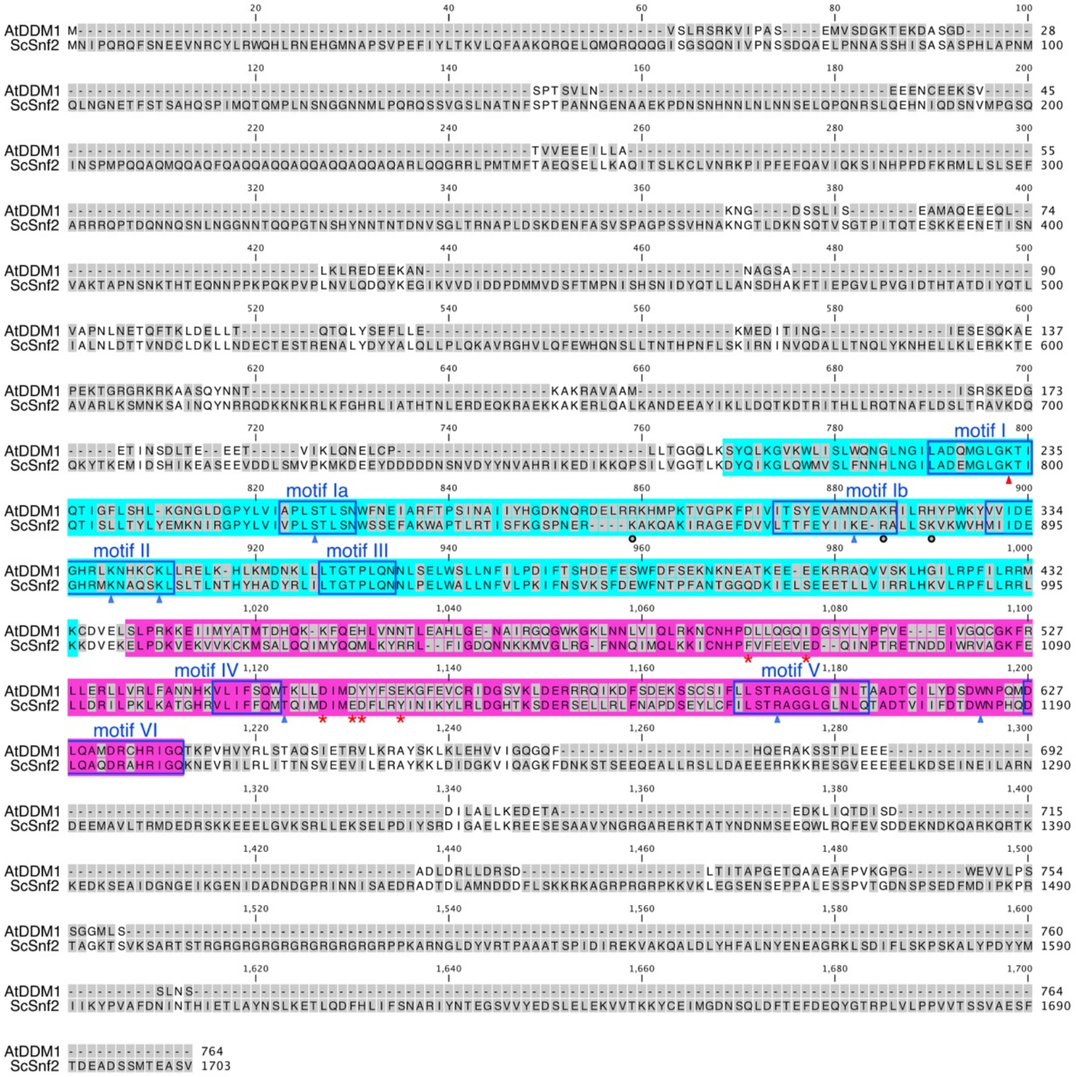

Supplementary Figure 8: Amino acid sequence alignment between AtDDM1 and ScSnf2.

The amino acid residues that differ between AtDDM1 and ScSnf2 are shown with a grey background. The amino acid residues corresponding to motifs I-VI identified in *Myceliophthora thermophila* Snf2[2] are shown in blue boxes. Cyan and magenta boxes correspond to ATPase core domains 1 and 2, respectively. The red arrowhead indicates the residue involved in nucleotide binding, discussed in previous studies[3-5]. Blue arrowheads and black circles indicate residues involved in primary and secondary DNA binding, respectively, as discussed previously[6]. Red asterisks indicate residues that may be involved in binding to the N-terminal tail of H4, as shown in Figure 7c and Supplementary Figure 13b.

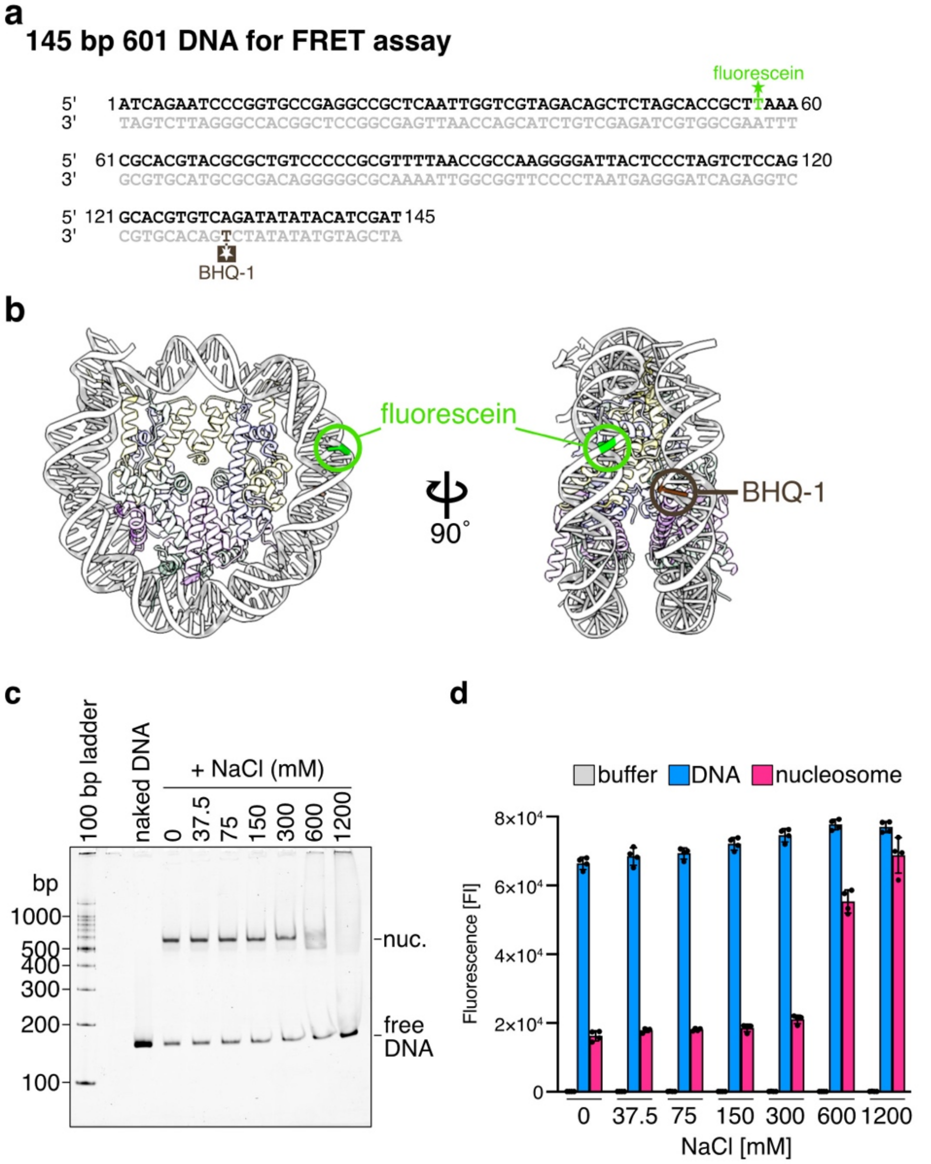

Supplementary Figure 9: Preparation of nucleosomes for FRET assay.

a, Sequence of the DNA fragment used for nucleosome reconstitution. The bases fused with fluorescein and its quencher (BHQ-1) are colored green and brown, respectively. **b,** Cryo-EM structures of the AtH2A.W nucleosome lacking the 4 base-pair linker DNA, with the locations of fluorescein and BHQ-1. **c,** Native-PAGE analysis for the disruption of nucleosomes by NaCl. **d,** Graphical presentation of the FRET assay with NaCl. Means and error bars represent SD from four independent experiments. Source data are provided as a Source Data file.

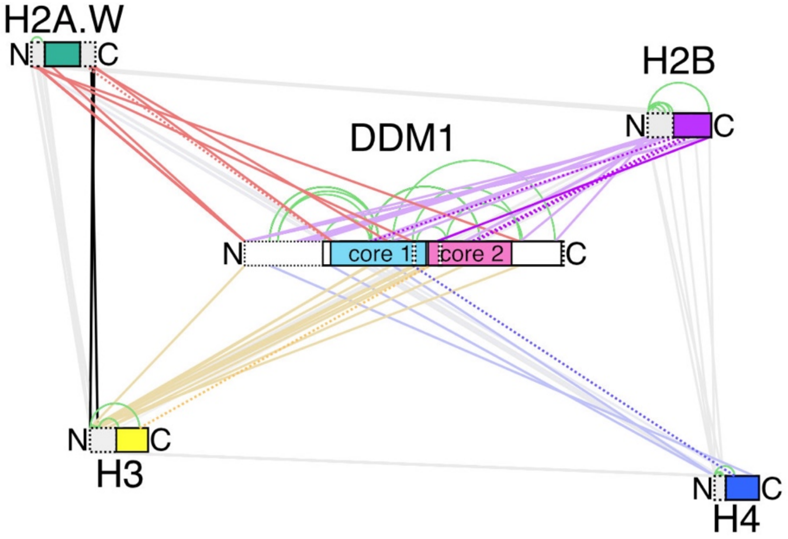

Supplementary Figure 10: Crosslinking mass spectrometry of DDM1-bound nucleosomes.

Schematic representation of the results obtained by crosslinking mass spectrometry of the DDM1-bound nucleosomes containing AtH2A.W. Grey (between histones), black (between AtH2A.W and AtH3), red (between AtH2A.W and AtDDM1), purple (between AtH2B and AtDDM1), yellow (between AtH3 and AtDDM1), and blue (between AtH4 and AtDDM1) lines correspond to inter-protein crosslinks, where faint colored lines indicate the interactions between disordered regions. Dashed lines indicate the crosslinking peptides, in which the distances between peptides were longer than the crosslinking areas based on our cryo-EM structure of the AtDDM1-AtH2A.W nucleosome complex. Dashed boxes indicate the disordered regions in the cryo-EM structure of the AtDDM1-AtH2A.W nucleosome complex. Green lines correspond to intra-protein crosslinks.

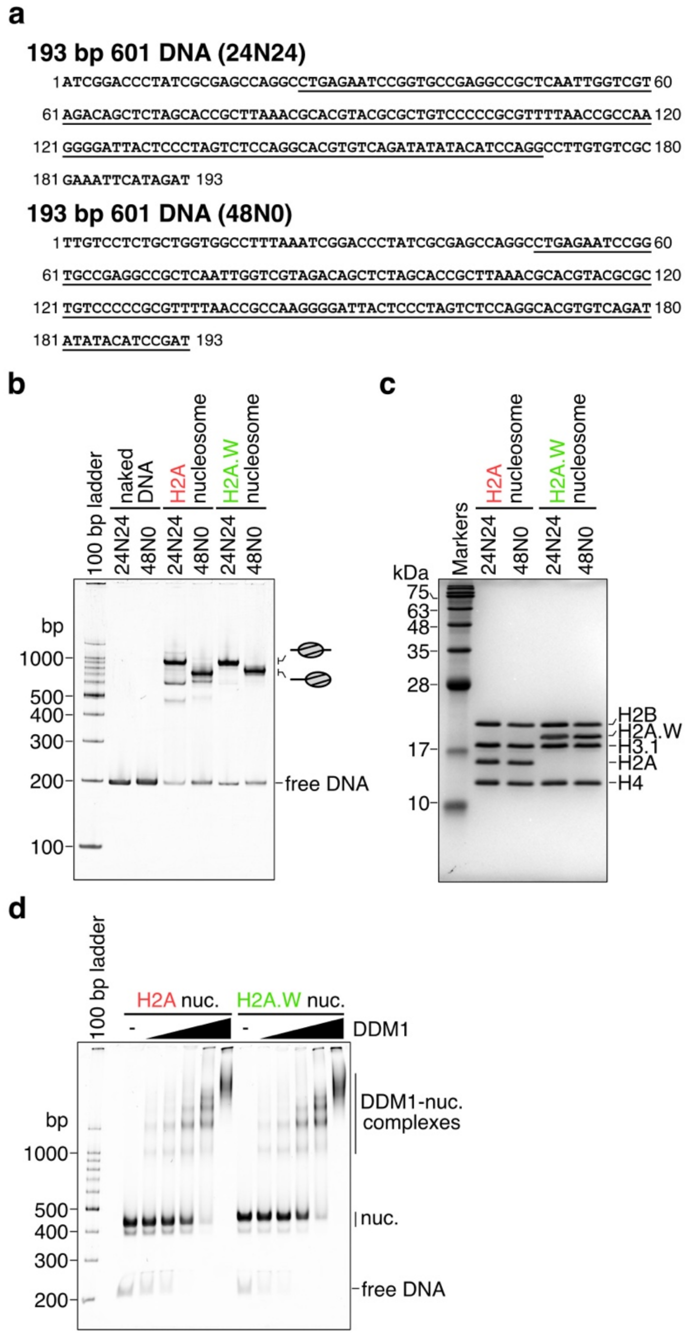

Supplementary Figure 11: Preparation of nucleosomes for nucleosome sliding assay.

a, Sequence of the DNA fragment used for nucleosome reconstitution. The Widom 601 positioning sequence is underlined. **b and c,** Native-PAGE (**b**) and SDS-PAGE (**c**) analyses of purified nucleosomes containing AtH2A and AtH2A.W. **d,** Native-PAGE analyses of the electrophoresis mobility shift assay for the binding between AtDDM1 and 48N0 nucleosomes.

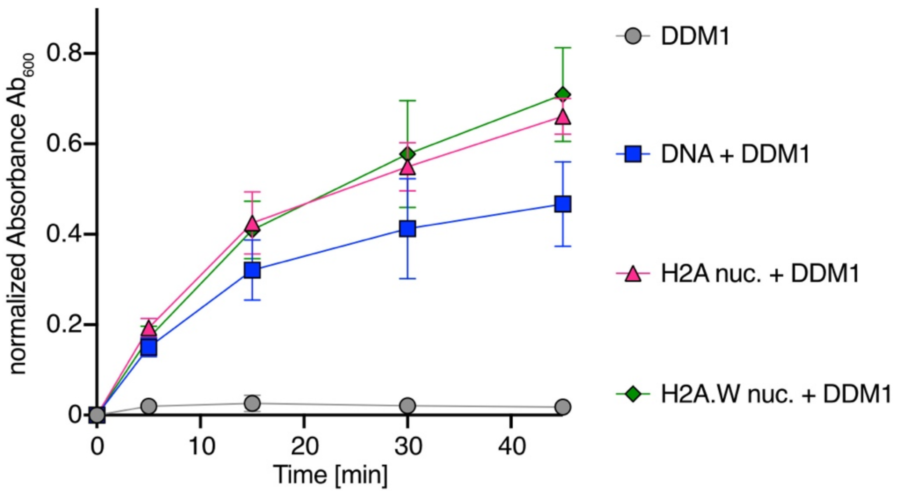

Supplementary Figure 12: ATPase assay.

Graphical presentation of the ATPase assay results with AtDDM1. Means and error bars represent SD from four independent experiments. Source data are provided as a Source Data file.

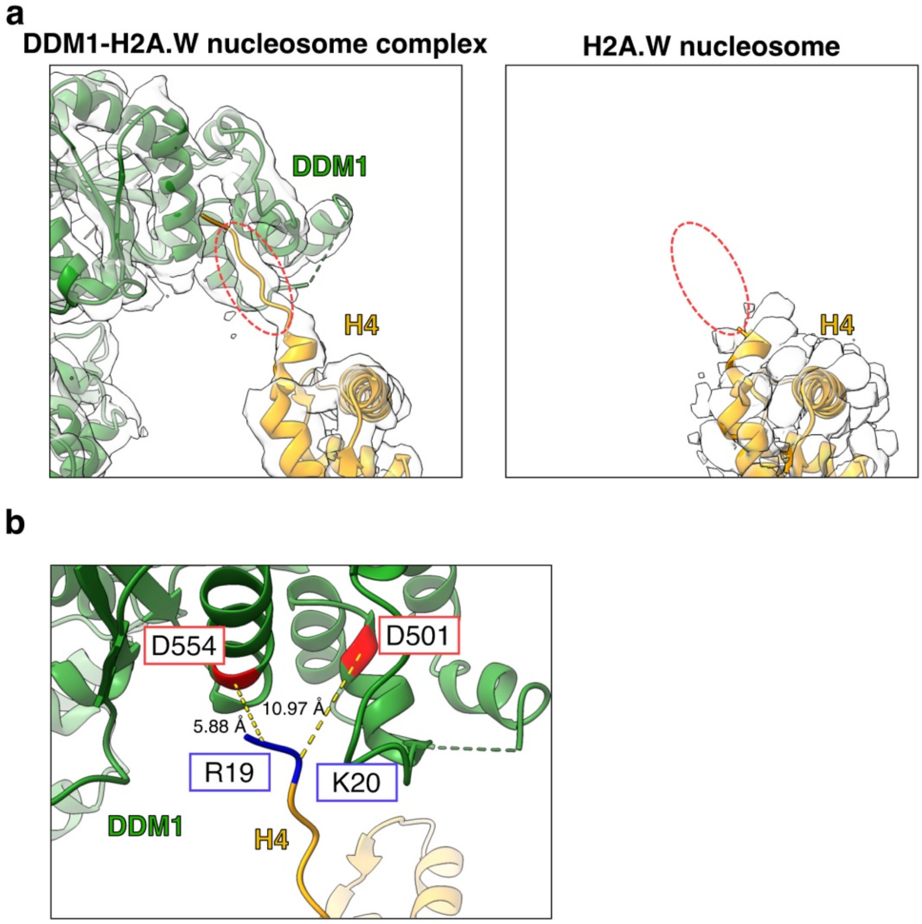

Supplementary Figure 13: Map to density figures of the N-terminal tail of H4 in the AtDDM1-bound AtH2A.W nucleosome and the AtH2A.W nucleosome.

a, Cryo-EM maps for the H4 N-terminal tail of the AtDDM1-AtH2A nucleosome complex (left) and the AtH2A.W nucleosome (right), respectively. Dashed red circles indicate the H4 N-terminal tail disordered in the AtH2A.W nucleosome. **b,** Close-up view of the possible interaction between AtDDM1 and the H4 N-terminal tail. The distances between the Cα atoms of possibly interacting residues are shown.

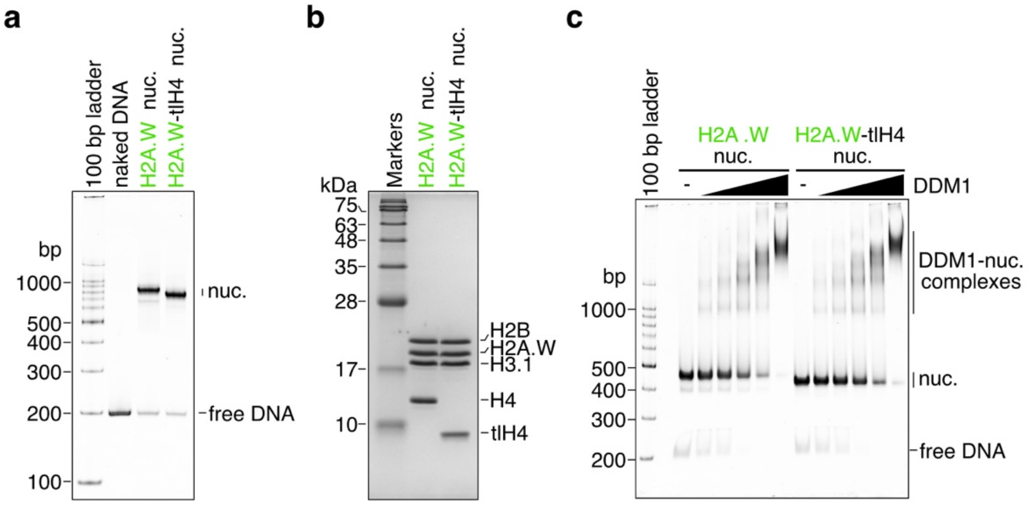

Supplementary Figure 14: Preparation of nucleosomes lacking the N-terminal tail of AtH4.

a and b, Native-PAGE (**a**) and SDS-PAGE (**b**) analyses of purified 48N0 nucleosomes containing H2A.W, with or without the N-terminal tail of AtH4. **c,** Native-PAGE analyses of the electrophoresis mobility shift assay for the binding between AtDDM1 and 48N0 nucleosomes.

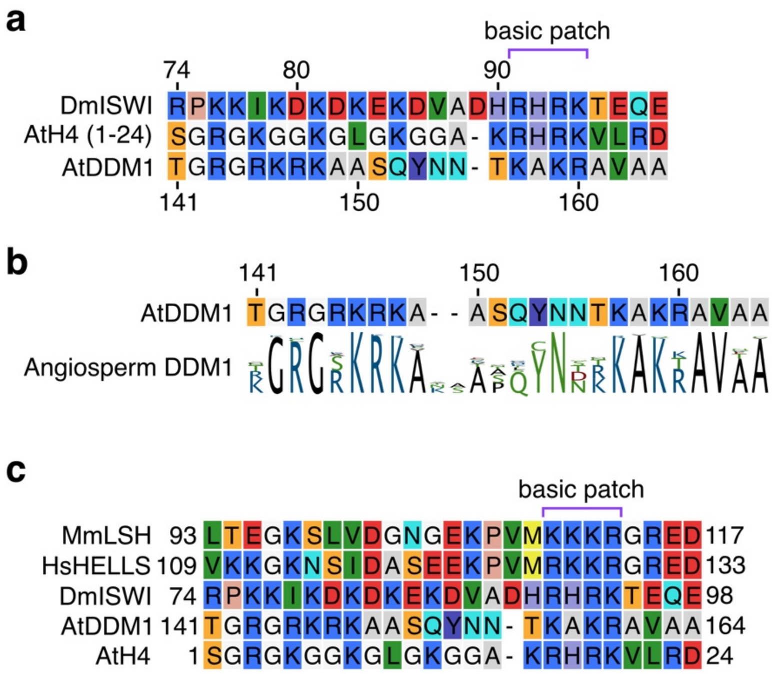

Supplementary Figure 15: Amino acid sequence alignment of putative AutoN regions of AtDDM1.

a, Amino acid sequence alignment of the *Drosophila* ISWI AutoN region, the N-terminal tail of AtH4, and AtDDM1. The basic patch defined previously[7,8] is shown with a purple bracket. **b,** Sequence logo of alignments of DDM1s from flowering plants (angiosperms)[3] with the sequence from AtDDM1. **c,** Amino acid sequence alignment of the *Drosophila* ISWI AutoN region, the N-terminal tail of AtH4, and AtDDM1. The basic patch defined previously is shown with a purple bracket.

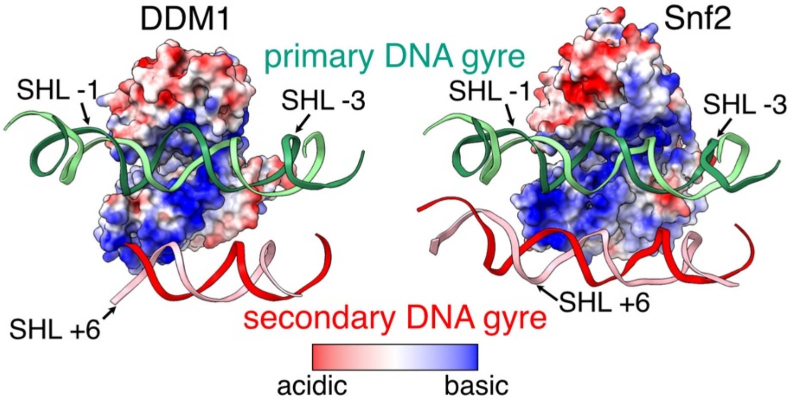

Supplementary Figure 16: The structures of nucleosomal DNA at SHL -2 and +6. Electrostatic potentials of the atomic surfaces of DDM1 (left) and ScSnf2 (right, PDB ID: 5X0Y[6] [https://www.rcsb.org/structure/5x0y] (Snf2-nucleosome complex)), which face toward the primary and secondary DNA gyres.

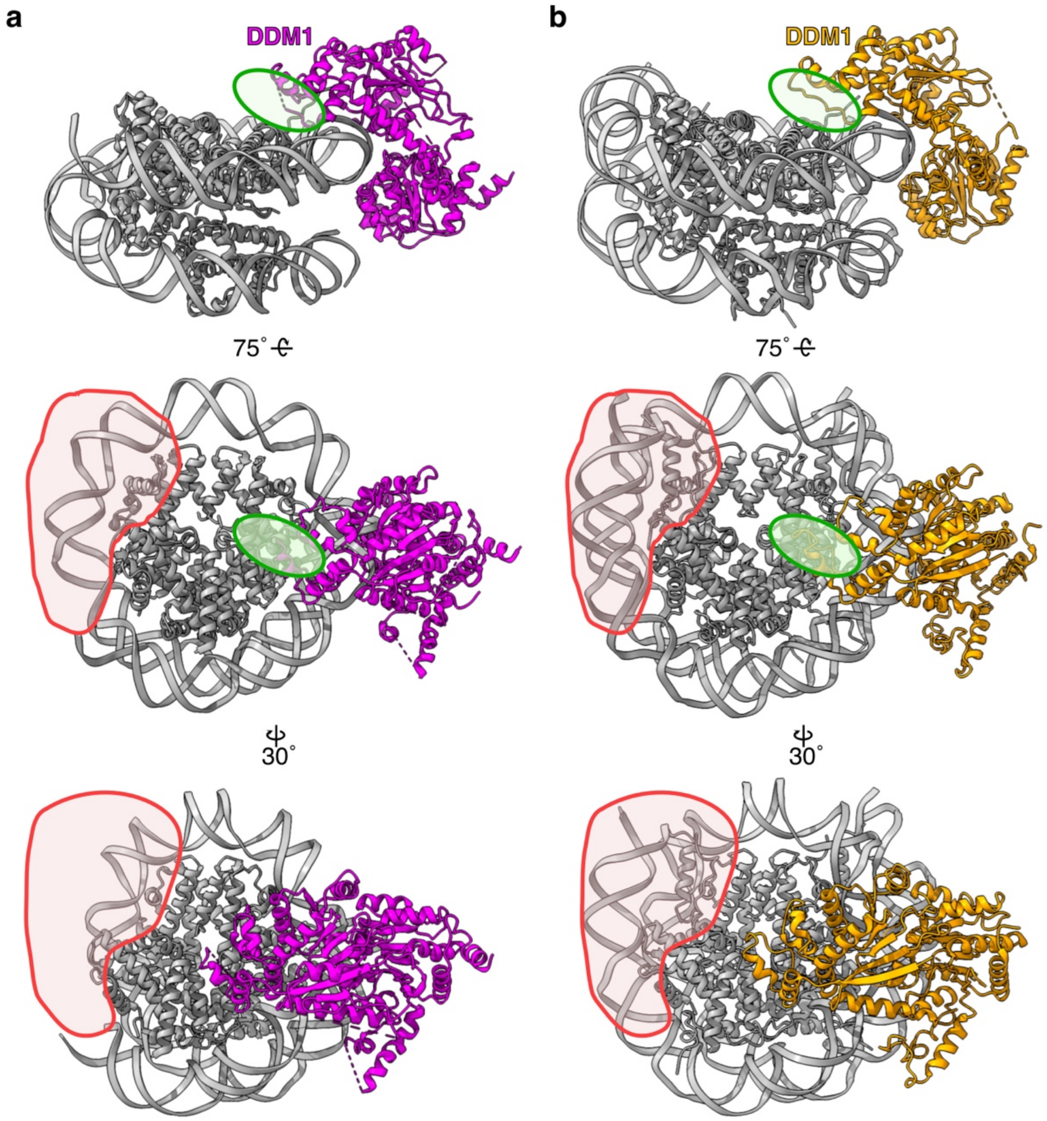

Supplementary Figure 17: Structural comparison of the AtDDM1-nucleosome complexes from the current and previous studies[9].

a and b, Cryo-EM structures of the AtDDM1-AtH2A.W nucleosome from the current (**a**) and previous (right, PDB ID: 7UX9[9] [https://www.rcsb.org/structure/7UX9] (DDM1-nucleosome complex)) (**b**) studies. Green areas indicate the loop regions of AtDDM1 that contact the H3 molecules identified in the previous study. Red areas indicate the disordered regions identified in the current study.

SUPPLEMENTARY REFERENCES

1. Tan, Y. Z. *et al.* Addressing preferred specimen orientation in single-particle cryo-EM through tilting. *Nat Methods* **14**, 793–796 (2017).
2. Xia, X., Liu, X., Li, T., Fang, X. & Chen, Z. Structure of chromatin remodeler Swi2/Snf2 in the resting state. *Nat Struct Mol Biol* **23**, 722–9 (2016).
3. Osakabe, A. *et al.* The chromatin remodeler DDM1 prevents transposon mobility through deposition of histone variant H2A.W. *Nat Cell Biol* **23**, 391–400 (2021).
4. Li, M. *et al.* Mechanism of DNA translocation underlying chromatin remodelling by Snf2. *Nature* **567**, 409–413 (2019).
5. Linder, P. & Jankowsky, E. From unwinding to clamping - the DEAD box RNA helicase family. *Nat Rev Mol Cell Biol* **12**, 505–16 (2011).
6. Liu, X., Li, M., Xia, X., Li, X. & Chen, Z. Mechanism of chromatin remodelling revealed by the Snf2-nucleosome structure. *Nature* **544**, 440–445 (2017).
7. Clapier, C. R. & Cairns, B. R. Regulation of ISWI involves inhibitory modules antagonized by nucleosomal epitopes. *Nature* **492**, 280–4 (2012).
8. Ludwigsen, J. *et al.* Concerted regulation of ISWI by an autoinhibitory domain and the H4 N-terminal tail. *Elife* **6**, (2017).
9. Lee, S. C. *et al.* Chromatin remodeling of histone H3 variants by DDM1 underlies epigenetic inheritance of DNA methylation. *Cell* **186**, 4100-4116.e15 (2023).
