# Supplementary material for: Molecular and structural basis of the chromatin remodeling activity by Arabidopsis DDM1
Source: Nat Commun. 2024 Jul 11;15:5187. doi: 10.1038/s41467-024-49465-w (PMC11239853; doi:10.1038/s41467-024-49465-w)
Supplement: Supplementary file 2 — Reporting Summary [file 41467_2024_49465_MOESM2_ESM.pdf]

nature portfolio

Corresponding author(s): Akihisa Osakabe

Last updated by author(s): May 31, 2024

Reporting Summary

Nature Portfolio wishes to improve the reproducibility of the work that we publish. This form provides structure for consistency and transparency in reporting. For further information on Nature Portfolio policies, see our Editorial Policies and the Editorial Policy Checklist.

Statistics

For all statistical analyses, confirm that the following items are present in the figure legend, table legend, main text, or Methods section.

n/a	Confirmed	
☐	☒	The exact sample size (*n*) for each experimental group/condition, given as a discrete number and unit of measurement
☐	☒	A statement on whether measurements were taken from distinct samples or whether the same sample was measured repeatedly
☐	☒	The statistical test(s) used AND whether they are one- or two-sided<br>*Only common tests should be described solely by name; describe more complex techniques in the Methods section.*
☒	☐	A description of all covariates tested
☒	☐	A description of any assumptions or corrections, such as tests of normality and adjustment for multiple comparisons
☐	☒	A full description of the statistical parameters including central tendency (e.g. means) or other basic estimates (e.g. regression coefficient) AND variation (e.g. standard deviation) or associated estimates of uncertainty (e.g. confidence intervals)
☒	☐	For null hypothesis testing, the test statistic (e.g. *F*, *t*, *r*) with confidence intervals, effect sizes, degrees of freedom and *P* value noted<br>*Give P values as exact values whenever suitable.*
☒	☐	For Bayesian analysis, information on the choice of priors and Markov chain Monte Carlo settings
☒	☐	For hierarchical and complex designs, identification of the appropriate level for tests and full reporting of outcomes
☒	☐	Estimates of effect sizes (e.g. Cohen's *d*, Pearson's *r*), indicating how they were calculated

Our web collection on statistics for biologists contains articles on many of the points above.

Software and code

Policy information about availability of computer code

Data collection	EPU (TheremoFisherScientific)
Data analysis	iBright Analysis Software version 5.2.0, xQuest/xProphet version 2.1.5, UCSF Chimera X 1.5, CLC Main Workbench version 21.0.5, Relion4, MotionCor2 1.4, ctffind-4.1, ISOLDE 1.4, Coot 0.9.8.8, PHENIX 1.19.2, and Prism 10.2.1.

For manuscripts utilizing custom algorithms or software that are central to the research but not yet described in published literature, software must be made available to editors and reviewers. We strongly encourage code deposition in a community repository (e.g. GitHub). See the Nature Portfolio guidelines for submitting code & software for further information.

Data

Policy information about availability of data

All manuscripts must include a data availability statement. This statement should provide the following information, where applicable:

- Accession codes, unique identifiers, or web links for publicly available datasets
- A description of any restrictions on data availability
- For clinical datasets or third party data, please ensure that the statement adheres to our policy

Cryo-EM maps and atomic models were deposited in EMDB (https://www.ebi.ac.uk/emdb/) and PDB (https://www.rcsb.org/) as EMD-36083 [https://www.ebi.ac.uk/emdb/EMD-36083] (EMDB) and 8J90 [https://www.wwpdb.org/pdb?id=pdb_00008j90] (PDB) for the AtDDM1-nucleosome complex, EMD-36084 [https://www.ebi.ac.uk/emdb/EMD-36084] (EMDB) and 8J91 [https://www.wwpdb.org/pdb?id=pdb_00008j91] (PDB) for the nucleosome containing AtH2A, and

EMD-36085 [https://www.ebi.ac.uk/emdb/EMD-36085] (EMDB) and 8J92 [https://www.wwpdb.org/pdb?id=pdb_00008j92] (PDB) for the nucleosome containing AtH2A.W. Raw mass spectrometry data in this study was deposited to the proteomeXchange Consortium (PXD043417 [https://proteomecentral.proteomexchange.org/cgi/GetDataset?ID=PXD043417]) via the Japan ProteOme STandard (JPOST) repository (https://repository.jpostdb.org/preview/35449496764b61cff0ceb1). The raw mass spectrometry data (JPST002218 [https://repository.jpostdb.org/entry/JPST002218]) can be accessed in JPOST repository. The structures of the nucleosome composed of the Widom 601 DNA sequence, human nucleosome core particle, Snf2-nucleosome complex, and DDM1-nucleosome complex used in this study can be found in the Protein Data Bank under the accession codes 3LZ0 [https://www.rcsb.org/structure/3lz0] , 7VZ4 [https://www.rcsb.org/structure/7VZ4], 5X0Y [https://www.rcsb.org/structure/5x0y], and 7UX9 [https://www.rcsb.org/structure/7UX9], respectively.

Research involving human participants, their data, or biological material

Policy information about studies with human participants or human data. See also policy information about sex, gender (identity/presentation), and sexual orientation and race, ethnicity and racism.

Reporting on sex and gender	n/a
Reporting on race, ethnicity, or other socially relevant groupings	n/a
Population characteristics	n/a
Recruitment	n/a
Ethics oversight	n/a

Note that full information on the approval of the study protocol must also be provided in the manuscript.

Field-specific reporting

Please select the one below that is the best fit for your research. If you are not sure, read the appropriate sections before making your selection.

☒ Life sciences ☐ Behavioural & social sciences ☐ Ecological, evolutionary & environmental sciences

For a reference copy of the document with all sections, see nature.com/documents/nr-reporting-summary-flat.pdf

Life sciences study design

All studies must disclose on these points even when the disclosure is negative.

Sample size	The sample size was based on similar studies in field. For all nucleosome sliding assays, FRET and ATPase assay, and restriction enzyme susceptibility assay, we performed three, four, and five independent experiments, which were used to calculate the standard deviations. For cryo-EM analyses, we used 16,161 (AtDDM1-nucleosome), 6,520 (nucleosome containing AtH2A), and 5,570 (nucleosome containing AtH2A.W) micrographs for structure reconstitution, which were sufficient for structure reconstruction at the resolution required for the discussion in this paper.
Data exclusions	No data were excluded from the analyses.
Replication	The reproducibility for nucleosome sliding assay, FRET assay, ATPase assay, and restriction enzyme susceptibility assay was confirmed by at least three independent experiments.
Randomization	Randomization was not performed since it does not contain any animals or human participants. Randomization is not necessary for the discussion in this study.
Blinding	Biochemical and cryo-EM analyses are not blinded. Researchers' biases will not occur in our biochemical assays and cryo-EM structural analyses.

Reporting for specific materials, systems and methods

We require information from authors about some types of materials, experimental systems and methods used in many studies. Here, indicate whether each material, system or method listed is relevant to your study. If you are not sure if a list item applies to your research, read the appropriate section before selecting a response.

Materials & experimental systems

n/a	Involved in the study
☒	☐ Antibodies
☒	☐ Eukaryotic cell lines
☒	☐ Palaeontology and archaeology
☒	☐ Animals and other organisms
☒	☐ Clinical data
☒	☐ Dual use research of concern
☒	☐ Plants

Methods

n/a	Involved in the study
☒	☐ ChIP-seq
☒	☐ Flow cytometry
☒	☐ MRI-based neuroimaging

Plants

Seed stocks

We don't use any plant materials for this manuscript.

Novel plant genotypes

n/a

Authentication

n/a
